# The Medication of the Mouth through Dentifrices

**Published:** 1911-06-15

**Authors:** Wallace Seccombe


### THE MEDICATION OF THE MOUTH THROUGH DENTIFRICES.

BY WALLACE SECCOMBE.

A Dentifrice is primarily a cleansing agent. When it becomes something more than a cleanser it occupies dangerous ground. The medication of the mucous membrane of the mouth is a serious proceeding and one not to be undertaken lightly. The medication of healthy mouths is vicious in principle and dangerous in parctice. Medication through a dentifrice presupposes the universal presence of a diseased condition in the mouths of mankind, which, in every case, demands medicinal treatment in precisely the same way.

Beware the dentifrice fearfully and wonderfully made. Beware the paste or powder containing a myriad of ingredients. *Back to the simple formula* should be the profession's definite instruction to the manufacturer.

Wherein is the advantage of the stimulation of the salivary glands with a consequent abnormal flow of saliva? Chewing-gum manufacturers have long enjoyed the privilege of advertising such an advantage (?). The gum man continues to fool nature into thinking she is having a good square meal, which in reality is only rubber. But you can't fool nature all the time.

However the tooth paste man has risen to the occasion, and the manufactures of a certain Tooth Paste claim that it "excites an abundant flow of alkaline saliva, brought about by the Chlorate of Potash that it contains."

Here's a chance to make the saliva flow without even moving your jaws. It ought certainly to appeal to the tired or lazy individual who is suffering from inertia. Too bad the universal need of mankind is not "an increased flow of saliva"—what a boon this tooth paste would then be!

Among the claims made for this particular paste are these;

Excites circulation in the gums (tonic).

Stimulates glandular secretion.

Deodorizing agent.

Efficacious in tender or bleeding gums.

Efficacious in mercurial stomatitis.

Pyorrhea alveolaris is benefited and often cured.

Enemic and shrunken gums take on a healthier glow.

Swollen and spongy gums become hardened and healthy.

Preventive of dental caries and affections of the tonsils, including diphtheria.

Cleans and polishes the teeth and destroys bacteria lodged between them.

Thus we learn the action of this wonderful remedy for most all the ills to which the oral flesh of man is heir. But what will it do to the ordinary, every-day, healthy, normal mouth?

Frankly, we wouldn't hazard a guess!—*Oral Health.*

